# Evaluation of Hepatoprotective Potential of Polyherbal Preparations in CCl_4_-Induced Hepatotoxicity in Mice

**DOI:** 10.1155/2022/3169500

**Published:** 2022-02-27

**Authors:** Rayhana Begum, Sonia Akther Papia, Mst Marium Begum, Hongbin Wang, Rubaba Karim, Rebeka Sultana, Priyanka Rani Das, Taslima Begum, Md. Ragibul Islam, Nargis Manwar, Md. Sohanur Rahman

**Affiliations:** ^1^Department of Pharmacy, Primeasia University, Dhaka, Bangladesh; ^2^Department of Pharmacy, East West University, Aftabnagar, Dhaka-1212, Bangladesh; ^3^Department of Pharmaceutical and Biomedical Sciences, College of Pharmacy, California Northstate University, CA, USA; ^4^School of Molecular Medical Sciences, University of Nottingham, Nottingham, UK; ^5^Department of Biochemistry and Molecular Biology, Trust University, Barishal, Ruiya, Nobogram Road, Barishal 8200, Bangladesh

## Abstract

**Background:**

Polyherbal formulations (PLFs) have been widely used for liver protection, treatment for hepatic dysfunction, and regeneration. They can also enhance appetite and protect the gastrointestinal tract from injury. In spite of the prevalent use, there is a need of scientific evidence on their effectiveness and safety. The objective of the present study was to assess the hepatoprotective effect of polyherbal formulations (commercially available in Bangladesh namely Heptaliv, Holyliv, Icturn, and J-deenar) in CCl_4_-induced hepatotoxicity in mice.

**Methods:**

In this study, Swiss albino mice were treated for 7 days with distilled water or PLFs (2.6 and 5.2 ml/kg body weight/day, per os.) followed by single subcutaneous injection of CCl_4_ (1 ml/kg body weight, diluted with olive oil in 1 : 1 ratio) on day 8. Twenty-four hours after CCl_4_ administration, the mice were monitored for the effects of PLFs on liver morphology, biochemical parameters including serum aspartate transaminase (AST), serum alanine transaminase (ALT), alkaline phosphatase (ALP), and total bilirubin. Phenobarbitone-induced sleeping time and histopathology changes in liver tissues were also monitored.

**Results:**

CCl_4_ administration caused significant hepatotoxicity as evidenced by marked elevation in AST, ALT, ALP, and total bilirubin. Phenobarbitone-induced sleeping time and infiltration of inflammatory cells and centrizonal necrosis on histological examination of liver demonstrated hepatic injury after CCl_4_ administration. However, the administration of Icturn and J-deenar polyherbal formulations at the higher dose significantly decreased the levels of AST, ALT, ALP, and total bilirubin. Moreover, pentobarbitone-induced sleeping time and histopathological analysis also revealed significant improvement as result of treatment with formulations Icturn and J-deenar.

**Conclusion:**

Our results confirmed that polyherbal formulations (Icturn and J-deenar) can significantly prevent CCl_4_-induced hepatotoxicity in mice, demonstrating their protective effect for liver.

## 1. Background 

Liver injury can be caused by various undesirable chemical components like toxic chemicals (e.g. carbon tetrachloride, aflatoxin, thioacetamide, trichloroethylene, etc.), therapeutic agents (e.g. antibiotics, antitubercular drugs, paracetamol, aspirin, ibuprofen certain chemotherapeutic agents, etc.), microbial agents (e.g. hepatitis virus, leptospira, malarial parasites, etc.) obesity, genetic defects, autoimmune disorders, chronic consumption of alcohol, certain herbs, hormones (such as birth control pills), and anabolic steroids [1–3]. Hepatotoxicity is linked with cellular necrosis; elevated free radicals induced oxidative stress that can directly cause destruction of cell membrane with subsequent alteration in the metabolic pathways [4]. A significant role is played by the reactive oxygen species (ROS) in maintaining degenerative cellular changes that affect a number of major organs in our body like heart, liver, lung, and kidney [5]. Additionally, the mechanism of antioxidant defense system of our body is downregulated during liver injury. Several factors like radiations (e.g. ultraviolet radiation, X-rays etc.), pollutants, and endogenous metabolites are responsible for generation of free radicals in our cells. CCl_4_ is considerably applied to induce liver toxicity in a variety of experimental models since it is subsequently suitable agent for inducing human cirrhosis-like effect in animal models [5, 6]. CCL_4_-induced hepatic damage is brought through the production of free radicals like CCl_3_ • and CCl_3_OO• along with cytochrome P450 of liver microsomes. These free radicals are thought to interact with membrane lipids causing their peroxidation [5]. Membrane degeneration of hepatocytes with subsequent generation of aspartate transaminase (AST), alanine transaminase (ALT), alkaline phosphatase (ALP), lactate dehydrogenase (LDH), and *γ*-glutamyltransferase (*γ*-GT), the marker enzymes of hepatotoxicity, centrilobular necrosis, and steatosis, are the consequences of CCl_4_-induced lipid peroxidation [5].

Almost all the allopathic medicines are being used to treat hepatotoxicity in which certain drugs like cholestyramine, ursodeoxycholic acid, spironolactone, loratadine, vasopressin, etc. have side effects including diarrhea, constipation, flatulence, abdominal discomfort, variable response and may cause encephalopathy in severe cases [1, 7]. Due to numerous undesirable effects caused by the modern hepatoprotective drugs in allopathic medical practices, several medicinal plants and their formulations are being extensively used as more trustworthy therapeutics for liver disorders in ethnomedical practices and in traditional medicine system in India, Africa, and elsewhere [8, 9]. These drugs contain phytochemical constituents like phenol, coumarins, monoterpenes, glycosides, flavonoids, alkaloids, and xanthenes, which play a noteworthy role in hepatoprotective activity [10]. Four billion people (about 80% of global population) in the developing countries depend on herbal medicinal products to treat many ailments and the use of herbal remedies has enhanced worldwide over recent years [11–14]. In Bangladesh, 85% of the total population live in rural areas [15], and about 80% are dependent on herbal medication for their primary healthcare [16, 17] due to its fewer side effects as well as lower cost [18].

In the traditional medicine, plant extract formulation and combined extract of plants are used as therapeutics rather than single drug. There is a growing awareness in polyherbal formulation to treat various diseases including liver diseases worldwide and a good number of herbal formulations are well accepted for their hepatoprotective effect [19]. About 600 commercially available herbal formulations in the market all over the world that claim to possess hepatoprotective effect [20]. As additional new herbal medicinal products are brought into the market, public health issues and alarms regarding their safety are also extremely demanded. Nevertheless, some herbal formulations are potentially effective for the treatment of various diseases and are comprehensively used while many of them remain untested and their use also not continuously monitored [14]. Thus, the absence of knowledge regarding their potential adverse effects and identification of safe and effective therapies making it complex to evaluate the promotion of their rational use [21]. However, herbal formulations efficacy should be tested by standard investigational methods including in vivo and in vitro studies to support their therapeutic claim. According to previous report, polyherbal formulations demonstrated curative effect on liver diseases and disorders in animal models [22]. Moreover, many plants and natural medicinal products beforehand showed protective effects against CCl4-induced liver toxicity [23–25]. Although there is availability of documents about individual polyherbal formulations, their relative evaluation is not adequately found in the literature.

In the present study, four marketed hepatoprotective polyherbal formulations (PLFs), namely Heptaliv, Holyliv, Icturn, and J-Deenar, marketed in Bangladesh, were selected to evaluate their effect in CCl_4_-induced hepatotoxic mice models. The objective of our present study is to evaluate the relative hepatoprotective effects of the above mentioned four polyherbal formulations based on the biochemical parameters and histopathology of the liver. Furthermore, phenobarbital-induced sleeping time (PST) was assessed to explore the physiological function of liver. It was evidenced that phenobarbital is generally metabolized through cytochrome p450 enzyme system in the liver. Drugs/chemicals that inhibit the cytochrome p450 enzyme will extend the duration of phenobarbital-induced sleeping time (PST) and vice versa [26].

## 2. Materials and Methods

### 2.1. Experimental Animals

Swiss albino mice (25–30 g, female, source of mice: Pharmacy Department of Jahangirnagar University, Dhaka Bangladesh) were used for the experiments. The animals were accommodated under standard environmental conditions (12 : 12 hr L : D cycle and 25 ± 2°C) with free access to standard food pellets and tap water *ad libitum* throughout the experimental period. All animals were subjected to euthanasia prior to surgery. The surgical procedure was carried out by anesthesia with 5% isoflurane in the presence of 100% oxygen. Animal handling and care for the present research was maintained according to the guidelines of National Institution of Health (Guide for the Care and Use of Laboratory Animals, NIH publication No: 85–23, revised in 1985). The present protocol was approved by the Biomedical Research Center, University of Dhaka, Bangladesh (Ref. No. BMRC/EC/2016–17/97).

### 2.2. Drugs and Chemicals

In the present study, four polyherbal hepatoprotective formulations including Heptaliv, Holyliv, Icturn, and J-deenar were randomly selected from the local markets (Table 1). These formulations were selected on the basis of the following claims by their manufacturers: (a) ayurvedic medicine, (b) hepatoprotective activity, and (c) sufficient shelf life. The formulations are also commercially available in liquid forms for easy administration. CCl_4_ and phenobarbitone were procured from Merck, Germany, and Sigma Aldrich Chemicals, India, respectively. Rest of the chemical reagents and enzyme assay kits were obtained from Merck (Darmstadt, Germany).

### 2.3. Dose Calculations of Polyherbal Formulations

The doses of polyherbal formulations (PLFs) for mice were calculated based on clinical doses as mentioned by the guideline of manufacturing company. Dose conversion (human to animal) was done by utilizing a standard conversion table [27]. It was reported that a partial hepatoprotective effects were observed by using 15 ml thrice a day in clinic [28]. The formulations were 1 : 10 diluted before oral administration of mice once daily for seven days. Mice were pretreated with PLFs at the doses of 2.6 ml/kg/day and 5.2 ml/kg/day; per os (p.o.) before CCl_4_ challenge.

### 2.4. Study Design

Animals were divided into 20 groups. Among all the groups, 10 groups (*n* = 6 mice) were subjected for the evaluation of biochemical and histopathological parameters (Table 2), and another 10 groups (*n* = 3 mice) were used for measuring phenobarbitone-induced sleeping time (Table 3). Liver injury was induced in mice by administering CCl_4_ subcutaneously (s. c.) in the lower abdomen in a suspension of olive oil in the ratio 1 : 1 v/v at the dose of 1 ml CCl_4_/kg BW of each animal.

### 2.5. Measurement of Body Weight Variation

During the experimental period, weights of the mice were recorded on day 0 and on day 9 using a digital weighing balance.

### 2.6. Blood Collection

Twenty-four hours after CCl_4_ administration, blood was collected from mice by cardiac puncture. Cardiac puncture was carried out under general anesthesia with 5% isoflurane in the presence of 100% oxygen. The serum was collected after the blood was centrifuged at 2500 rpm for 10 minutes. After blood collection, the animals were sacrificed by isoflurane overdose followed by cervical dislocation.

### 2.7. Biochemical and Histopathological Parameters (Groups 1–10)

The levels of aspartate transaminase (AST), alanine transaminase (ALT), alkaline phosphatase (ALP), and bilirubin were tested. Serum bilirubin was determined by using a standard method using alkaline methanolysis of bilirubin followed by chloroform extraction of bilirubin methyl esters [29]. The separation was done by chromatography and spectrophotometric determination at 430 nm [29]. Concentrations of serum aspartate transaminase (AST), serum alanine transaminase (ALT), and serum alkaline phosphatase (ALP) were determined by standard methods [30, 31]. Liver was excised and stored in 10% formalin fixative solution for histopathological evaluation. The histopathological evaluation was performed in Exim Bank Hospital, Department of histopathology, Dhaka, Bangladesh.

### 2.8. Phenobarbitone-Induced Sleeping Time (Groups 11–20)

After administration of phenobarbitone, the sleeping time (in minutes) of animals was recorded between sleep and rev up time. The present protocol for investigation of sleeping time was slightly altered by administering phenobarbitone in substitution of hexabarbitone [32]. In the present study, varying doses of phenobarbitone were given ranging from 20 to 60 mg/kg, ip. The dose of phenobarbitone used for this study was based on the earlier study by Girish C. [20]. Our preliminary experiments demonstrated that mice showed no sleep at the dose of 20 ml/kg, while prolonged sleeping time (>6 1/2 hours) was found at the dose of 60 mg/kg. Thus, 40 mg/kg (ip) dose was chosen to study the phenobarbitone-induced sleeping time (Table 3).

### 2.9. Statistical Analysis

All data were expressed as Mean ± S.E.M. and represented in tables. The results were statistically analyzed using one way analysis of variance (ANOVA) followed by Dunnett's test. The differences between the control and treatment groups were tested in 95% significance level (*p* < 0.05). All statistics have been done using GraphPad Instat version 3.10.

## 3. Results

### 3.1. Effect of Polyherbal Hepatoprotective Formulations on the Gain of Body Weight

In the carbon tetrachloride-induced hepatotoxicity method, the body weight of Group 2 was significantly decreased 24 h after CCl_4_ injection, but the body weights in Groups 4 to 10 were almost comparable to those in Group 1 (Table 4). Moreover, in the case of phenobarbitone-induced method, the drop in the body weight in the Group 12 was also observed, and Groups 13 to 20 were almost comparable to those in the Group 1 (Table 4).

### 3.2. Biochemical Assay

In the CCl_4_-treated group, the activity of AST (140.30 ± 5.9 IU/L), ALT (168.10 ± 2.9 IU/L), and ALP (34.40 ± 3.1 IU/L) was significantly higher (*p* < 0.01) in comparison to normal control (AST: 50.50 ± 2.3 IU/L, ALT: 30.70 ± 0.8 IU/L, ALP: 10.40 ± 0.5 IU/L). The serum bilirubin level was significantly increased (*p* < 0.01) in the CCl_4_-treated group compared to the normal level (3.1 ± 0.10 vs. 0.3 ± 0.01). At the end of the study, the three polyherbal formulations namely Holyliv, Heptaliv, and J-Deenar significantly reduced AST at the higher dose (5.2 ml/kg), whereas Ictrum demonstrated significant reduction in serum AST level at both doses (*p* < 0.05 and *p* < 0.01 at the doses of 2.6 ml/kg and 5.2 ml/kg, respectively) when compared with the CCl_4_ treated group (Table 5). Treatment with Heptaliv, Icturn, and J-Deenar at both doses caused a significant reduction (*p* < 0.01) in the activity of ALT compared to the CCl_4_-treated group. On the other hand, there was a significant increase in ALT level (*p* < 0.01) in group treated with Holyliv as compared to the CCl_4_-treated control group (Table 5). The groups treated with Heptaliv, Icturn, and J-Deena at the higher dose showed significant reduction in serum ALP level (*p* < 0.05) as compared to the CCl_4_-treated control group. Nevertheless, group treated with Holyliv at the both doses showed no significant reduction in serum ALP level as compared to the CCl_4_-treated control group (Table 5). There was a significant increase in bilirubin level in group treated with CCl_4_ as compared to the normal control group (*p* < 0.05). However, treatment with the formulations namely Holyliv, Heptaliv, and J-Deenar at dose of 5.2 ml/kg/day for 7 days prior to CCl_4_ intoxication showed significant decrease (*p* < 0.05) in level of bilirubin as compared to the CCl_4_ control group, while Ictrum demonstrated significant reduction in serum bilirubin level at the both doses (*p* < 0.05 and *p* < 0.01 at the doses of 2.6 ml/kg and 5.2 ml/kg, respectively) when compared with the CCl_4_ treated group (Table 5).

### 3.3. Effect of CCl_4_ and PLFs on Phenobarbitone-Induced Sleeping Time

The CCl_4_-induced liver injury directed to an elevation in the period of barbiturate-induced sleeping time (261 ± 5.7 min in normal control vs. 300 ± 13.3 min in the CCl_4_-induced hepatotoxicity group, *p* < 0.05). A notable restoration in the phenobarbitone-induced sleeping time (*p* < 0.01) was observed with all the polyherbal drugs at the higher dose. However, the effect of Holyliv-treated groups insignificant at the dose of 2.6 mg/kg (Table 6).

### 3.4. Histopathological Examination of Liver

**Figure 1:**
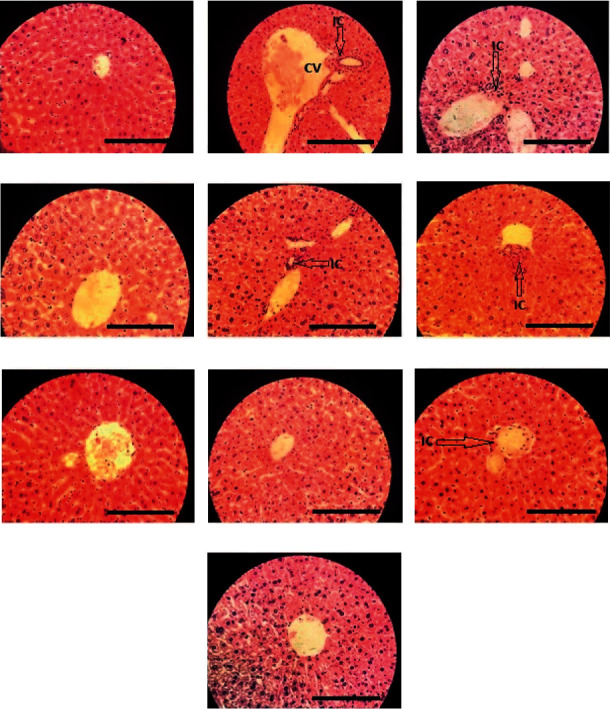
Histopathological changes in the liver. Each picture is a representative for each group as follows: (a) Normal control, (b) CCl4 control, (c) CCl4+ 2.6 ml/kg/day Holyliv, (d) CCl4+ 5.2 ml/kg/day Holyliv, (e) CCl4+ 2.6 ml/kg/day Heptaliv, (f) CCl4+ 5.2 ml/kg/day Heptaliv, (g) CCl4+ 2.6 ml/kg/day Icturn, (h) CCl4+ 5.2 ml/kg/day Icturn, (i) CCl4+ 2.6 ml/kg/day J-Deenar, (j) CCl4+ 5.2 ml/kg/day J-Deenar. Each group was assessed at 400X magnification, Scale bar: 40 *μ*m. CV = central vain, black arrow pointed the infiltration of inflammatory cells (IC).

While the characteristic architecture of liver tissue was observed with central vein in the healthy control (Figure 1(a)), liver tissue of CCl_4_-treated mice showed extensive necrosis, alteration in the array of cells around the central vein, and massive infiltration of inflammatory cells (Figure 1(b)). As shown in Figure 1, pretreatment with low dose of Holyliv and Heptaliv showed insignificant liver protection and numerous inflammatory cells infiltration (Figures 1(c) and 1(e), respectively). However, liver section of mice treated at the higher dose demonstrated moderate inflammatory cells infiltration (Figures 1(d) and 1(f)). On the other hand, pretreatment with low dose of Icturn and J-Deenar showed hepatic protection and moderate inflammatory cell infiltration (Figures 1(g) and 1(i), respectively). When mice were administered at the higher dose of Icturn and J-Deenar, they were found to significantly protect the liver from CCl_4_-induced liver damage as confirmed by restoration of a near typical architecture of the liver and minimal infiltration of inflammatory cells (Figures 1(h)and 1(j)).

## 4. Discussion

Our present study has evaluated hepatoprotective effects of four commercially available PLFs from Bangladesh market in CCl_4_-induced hepatotoxic mouse model. The results demonstrated that Icturn and J-Deenar at the higher dose (5.2 ml/kg/day) had better hepatoprotective effects than other PLFs. They could significantly restore level of serum biochemical markers (AST, ALT, ALP, and bilirubin) in mice treated with CCl_4_ to the level of normal mice.

Administration of CCl_4_ causes hepatopathy along with the significant elevation in AST, ALT, ALP and bilirubin in serum as compared with normal mice [33]. CCl_4_ is metabolized by the liver into highly reactive metabolites which either directly or indirectly cause lipid peroxidation of the hepatocytes [34]. Consistent lipid peroxidation causes cytosolic liver enzymes leak out of the swollen and necrotic hepatocytes into circulation. The elevated levels of enzymes and bilirubin are closely correlated to liver damages manifested by immense centrilobular necrosis, ballooning, degeneration and cellular infiltration of the liver [35]. Especially, ALT has been reported to be a precise marker of liver damage due to toxic drugs, alcohol and viral infection [36].

Icturn, a well-known commercial hepatoprotective PLF in Bangladesh, has been used as an effective treatment in liver diseases. Pretreatment with Icturn prevented the rise in liver enzyme parameters and kept them to near normal level when administered at 5.2 ml/kg BW/d dose in CCl_4_-induced hepatoxic mice. This may result from the effect of Icturn on the stabilization of plasma membrane as well as the repair of hepatic tissue damage caused by CCl_4_ toxicity. A previous study has demonstrated that PLFs have hepatoprotective activity like an active compound silymarin, a flavonolignan from *Silybum marianum* [28]. The protective functions of Icturn and J-Deenar as observed in the present study are in line with that study. Other formulations also showed appreciable activity, but the magnitude of effect was inferior to Icturn and J-Deenar. Moreover, Icturn was able to reduce the levels of enzymes more significantly indicating that it was more protective to hepatocytes and maintained normal liver physiology and also cause stabilization of plasma membrane and regeneration of damaged liver cells. The authors found that all the polyherbal formulation prevents the rise in ALT level except Holyliv formulation that might be attributed to the presence of xenobiotic(s) that instigated leakage of the enzyme into the blood via altered membrane permeability. No significant differences were observed when we compared with the protective effect of Icturn with that of J-Deenar.

Phenobarbitone is a hypnotic drug that is metabolized generally in liver. When it is given in mice that have damaged liver caused by CCl_4_ administration, it will cause longer mean duration of sleeping time as compared with mice with normal liver functions. Barbiturate will be poorly metabolized as a consequence of hepatic damage, which decreases in cytochrome P450s-mediated metabolic functional activity [20, 26]. Our resent study demonstrated that pretreatment with hepatoprotective PLFs prevented the changes of phenobarbitone-induced sleeping time in CCl_4_-treated mice. The results suggested that cytochrome P450s, which is the most critical xenobiotic metabolizing enzyme system in liver, are preserved by PLFs pretreatment in CCL_4_-challenged mice. It will be worthwhile to study the mechanisms how these PLFs protect cytochrome P450 in liver of CCl_4_-challenged mice.

Our histological study revealed that CCl_4_ treatment produced various changes in mouse hepatocytes, including centrizonal necrosis, cell inflammation, and infiltration of inflammatory cells. Pretreatment with Icturn and J-Deenar could prevent CCl_4_-induced changes in the hepatic structural design and protected the liver tissue from necrosis and degeneration (Figure 1). We speculate that PLFs generate hepatoprotective effects by preventing the toxic chemical reactions, which consequently generate oxidative stress, lipid peroxidation, and molecular changes that ultimately lead to liver tissue damages. Our data suggest that the hepatoprotective drugs may play a role in regeneration process, fibrosis prevention, or nodules formation, which may be expressed in the long-term use of the drug [37]. The improvement in histopathological findings with hepatoprotective PLFs was also found to be associated with the improvement in biochemical parameters.

Our study demonstrated a comparative investigation on possible use of four commercial polyherbal formulations of Bangladesh for the prevention of liver injury. Finally, our findings revealed that Icturn and J-Deenar at the higher dose had better hepatoprotective effects than other PLFs. However, the mechanism in relation to hepatoprotective effects needs to be investigated further under various preclinical conditions.

## 5. Conclusion

In conclusion, our study clearly demonstrated that PLFs Icturn and J-Deenar were the most effective in liver protection in CCL_4_-challenged mouse model, justifying their use as hepatoprotective agents in clinic. The efficacy of Heptaliv and Holyliv was low, and a dose adjustment may be necessary for their use in traditional medicine for human liver diseases.

## Figures and Tables

**Table 1:** The four marketed polyherbal formulations (liquid) used in current study of activities against liver injury (compiled from the manufacturer's instructions).

Formulations	Principal ingredients	Purpose	Standard dose
Heptaliv (S. B. Laboratories, Rjshahi, Bangladesh)	Each 5 ml extract contains *Aphanamixis polystachya* -1.52 gm, *Woodfordia floribunda*-0.24 gm, *Piper longum*-15.24 mg, *Zingiber officinale*-15.24 mg, Trifala-45.73 mg, and other ingredients	Jaundice, hepatic malfunction, liver inflammation, alcoholic liver disorder, liver cirrhosis, indigestion, constipation, and loss of appetite remedy	2–4 teaspoons 2 to 3 times daily.
Holyliv (GK pharma, station road, Tongi, Gazipur)	Each 5 ml extract contains *Cichorium intybus* root (400 mg), *Cichorium intybus* seed (200 mg), *Rose damascene* (200 mg), *Nymphaea alba* (100 mg), *Borago officinalis* (100 mg), *Cuscuta*, *reflexa* seed (150 mg), *Rheum emodi* (300 mg), and other ingredients.	Hepatitis, obstructive jaundice, edema, inflammation of uterus, pleurisy, constipation	2–3 teaspoons twice daily
Icturn (hamdard laboratories Bangladesh, Sonargaon, Narayangonj)	Each 5 ml extract contains *Cichorium endivia* root-400 gm, *Cichorium endivia* seed-200 mg, *Rose damascene-*200 mg, *Nympaea nouchali-*100 mg, *Borago officinalis*-100 mg, *Cuscuta*, *reflexa* seed-150 mg *Rheum emodi*-125 mg, and other ingredients.	Hepatitis, obstructive jaundice ascites constipation, alveolitis, pleurisy uterine inflammation, and metritis	2–3 teaspoons twice daily
J-deenar (janashastha pharmaceuticals, Dhaka, Bangladesh	Each 5 ml extract contains cuscuta leaf-400 mg, endive seeds-200 mg, male fern-200 mg, water lily-100 mg, borago gawjban-100 mg, cuscuta roots-150 mg, rhaw chine-125 mg and other ingredients	Hepatitis, jaundice, and constipation.	2 teaspoons 3 times daily after meal

**Table 2:** Dosage of orally administered drug in various groups in carbon tetrachloride-induced toxicity.

Groups	Treatment
Group 1	Normal control; the animals received distilled water.
Group 2	Pathological control; received distilled water for 7 days (p.o.) followed by a single dose of CCl_4_ (sc) on day 8
Group 3	Received holyliv formulation at 2.6 ml/kg BW/d for 7 days (p.o.) + a single dose of CCl_4_ on day 8
Group 4	Received holyliv formulation at 5.2 ml/kg BW/d for 7 days (p.o.) + a single dose of CCl_4_ on day 8
Group 5	Received heptaliv formulation at 2.6 ml/kg BW/d for 7 days (p.o.) + a single dose of CCl_4_ on day 8
Group 6	Received heptaliv formulation at 5.2 ml/kg BW/d for 7 days (p.o.) + a single dose of CCl_4_ on day 8
Group 7	Received icturn formulation at 2.6 ml/kg BW/d for 7 days (p.o.) + a single dose of CCl_4_ on day 8
Group 8	Received icturn formulation at 5.2 ml/kg BW/d for 7 days (p.o.) + a single dose of CCl_4_ on day 8
Group 9	Received J-Deenar formulation at 2.6 ml/kg BW/d for 7 days (p.o.) + a single dose of CCl_4_ on day 8
Group 10	Received J-Deenar formulation at 5.2 ml/kg BW/d for 7 days (p.o.) + a single dose of CCl_4_ on day 8

**Table 3:** Dosage of orally administered drug in various groups in phenobarbitone-induced sleeping time.

Groups	Treatment
Group 11	Normal control; received distilled water for 7 days (p.o.) + phenobarbitone single dose on day 9.
Group 12	Pathological control; distilled water for 7 days (p.o.) + CCl_4_ single dose on day 8 + phenobarbitone single dose on day 9.
Group 13	Received holyliv formulation at 2.6 ml/kg BW/d for 7 days (p.o.) + CCl_4_ single dose on day 8 + phenobarbitone single dose on day 9.
Group 14	Received holyliv formulation at 5.2 ml/kg BW/d for 7 days (p.o.) + CCl_4_ single dose on day 8 + phenobarbitone single dose on day 9.
Group 15	Received heptaliv formulation at 2.6 ml/kg BW/d for 7 days (p.o.) + CCl_4_ single dose on day 8 + phenobarbitone single dose on day 9.
Group 16	Received heptaliv formulation at 5.2 ml/kg BW/d for 7 days (p.o.) + CCl_4_ single dose on day 8 + phenobarbitone single dose on day 9.
Group 17	Received icturn formulation at 2.6 ml/kg BW/d for 7 days (p.o.) + CCl_4_ single dose on day 8 + phenobarbitone single dose on day 9.
Group 18	Received icturn formulation at 5.2 ml/kg BW/d for 7 days (p.o.) + CCl_4_ single dose on day 8 + phenobarbitone single dose on day 9.
Group 19	Received J-Deenar formulation at 2.6 ml/kg BW/d for 7 days (p.o.) + CCl_4_ single dose on day 8 + phenobarbitone single dose on day 9.
Group 20	Received J-Deenar formulation at 5.2 ml/kg BW/d for 7 days (p.o.) + CCl_4_ single dose on day 8 + phenobarbitone single dose on day 9.

**Table 4:** Effect of Polyherbal formulations on the gain of body weight.

CCl_4_-induced hepatotoxicity method		CCl_4_+ phenobarbitone-induced method	
Groups	Change in body weight (g)	Groups	Change in body weight (g)

Group-1	1.50 ± 0.08	Group-11	1.80 ± 0.07
Group-2	0.80 ± 0.03	Group-12	0.93 ± 0.06
Group-3	1.20 ± 0.05	Group-13	1.27 ± 0.07
Group-4	1.29 ± 0.06	Group-14	1.39 ± 0.05
Group-5	1.23 ± 0.07	Group-15	1.30 ± 0.08
Group-6	1.28 ± 0.04	Group-16	1.39 ± 0.09
Group-7	1.37 ± 0.05	Group-17	1.37 ± 0.07
Group-8	1.45 ± 0.07	Group-18	1.44 ± 0.08
Group-9	1.25 ± 0.08	Group-19	1.45 ± 0.08
Group-10	1.35 ± 0.06	Group-20	1.57 ± 0.07

Each value is Mean ± S.E.M (*n* = 3). Group 4 = CCl_4_+ 5.2 ml/kg/day Holyliv, Group 5 = CCl_4_+ 2.6 ml/kg/day Heptaliv, Group 6 = CCl_4_+ 5.2 ml/kg/day Heptaliv, Group 7 = CCl_4_+ 2.6 ml/kg/day Icturn, Group 8 = CCl_4_+ 5.2 ml/kg/day Icturn, Group 9 = CCl_4_+ 2.6 ml/kg/day J-Deenar, Group 10 = CCl_4_+ 5.2 ml/kg/day J-Deenar. Group 11 = normal control, Group 12 = CCl_4_+ phenobarbitone control, Group 13 = CCl_4_+ phenobarbitone + 2.6 ml/kg/day Holyliv, Group 14 = CCl_4_+ phenobarbitone + 5.2 ml/kg/day Holyliv, Group 15 = CCl_4_ + phenobarbitone +2.6 ml/kg/day Heptaliv, Group 16 = CCl_4_+ phenobarbitone + 5.2 ml/kg/day Heptaliv, Group 17 = CCl_4_+ phenobarbitone + 2.6 ml/kg/day Icturn, Group 18 = CCl_4_+ phenobarbitone + 5.2 ml/kg/day Icturn, Group 19 = CCl_4_+ phenobarbitone + 2.6 ml/kg/day J-Deenar, Group 20 = CCl_4_+ phenobarbitone + 5.2 ml/kg/day J-Deenar.

**Table 5:** Effect of polyherbal hepatoprotective formulations on biochemical serum parameters in CCl_4_-induced hepatotoxicity.

Groups	Liver enzymes			Bilirubin (mg/dl)
	AST (IU/L)	ALT (IU/L)	ALP (IU/L)	
Group 1	50.50 ± 2.30	30.70 ± 0.80	10.40 ± 0.50	0.30 ± 0.01
Group 2	140.30 ± 5.90^a^^∗∗^	168.10 ± 2.90^a^^∗∗^	34.40 ± 3.10^a^^∗∗^	3.10 ± 0.10^a^^∗∗^
Group 3	130.40 ± 3.90^b*η*^	298.20 ± 3.10^b^^∗∗^	33.70 ± 3.80^b*η*^	0.90 ± 0.07^b*η*^
Group 4	118.49 ± 4.20^b^^*∗*^	414.40 ± 4.70^b^^∗∗^	31.20 ± 2.00^b*η*^	0.50 ± 0.07^b^^*∗*^
Group 5	127.40 ± 4.10^b*η*^	80.10 ± 3.50^b^^∗∗^	27.30 ± 2.70^b*η*^	1.00 ± 0.06^b*η*^
Group 6	119.60 ± 4.80^b^^*∗*^	51.30 ± 3.70^b^^∗∗^	25.10 ± 2.10^b^^*∗*^	0.70 ± 0.08^b^^*∗*^
Group 7	121.36 ± 5.90^b^^*∗*^	65.12 ± 4.30^b^^∗∗^	26.40 ± 2.70^b*η*^	0.70 ± 0.06^b^^*∗*^
Group 8	77.00 ± 3.50^b^^∗∗^	35.50 ± 3.80^b^^∗∗^	21.80 ± 2.30^b^^∗∗^	0.50 ± 0.03^b^^∗∗^
Group 9	123.50 ± 3.80^b*η*^	75.55 ± 2.70^b^^∗∗^	27.00 ± 2.00^b*η*^	1.00 ± 0.07^b*η*^
Group 10	80.60 ± 3.20^b^^∗∗^	49.20 ± 2.40^b^^∗∗^	24.30 ± 2.20^b^^*∗*^	0.70 ± 0.06^b^^*∗*^

Each value is Mean ± S.E.M (*n* = 3). (^*∗*^) designates statistically significant difference from the respective group, using Dunnett's multiple comparison test (^∗∗^*p* < 0.01). (*η*) designates statistically no significant difference from the respective group using Dunnett's multiple comparison test (*p* > 0.05). Group 1 = normal control, Group 2 = CCl_4_ control, Group 3 = CCl_4_+ 2.6 ml/kg/day Holyliv, Group 4 = CCl_4_+ 5.2 ml/kg/day Holyliv, Group 5 = CCl_4_+ 2.6 ml/kg/day Heptaliv, Group 6 = CCl_4_+ 5.2 ml/kg/day Heptaliv, Group 7 = CCl_4_+ 2.6 ml/kg/day Icturn, Group 8 = CCl_4_+ 5.2 ml/kg/day Icturn, Group 9 = CCl_4_+ 2.6 ml/kg/day J-Deenar, Group 10 = CCl_4_+ 5.2 ml/kg/day J-Deenar.

**Table 6:** Effect of pretreatment with polyherbal formulations on phenobarbitone-induced sleeping time of mice in CCl_4_-induced hepatotoxicity.

Group	Phenobarbitone induced sleeping time (min)
Group 11	261.9 ± 5.7
Group 12	300.5 ± 10.3^a^^*∗*^
Group 13	268.3 ± 9.8^b*η*^
Group 14	225.7 ± 7.5^b^^∗∗^
Group 15	260.5 ± 8.1^b^^*∗*^
Group 16	196.8 ± 7.0^b^^∗∗^
Group 17	220.7 ± 8.1^b^^∗∗^
Group 18	188.4 ± 7.9^b^^∗∗^
Group 19	230.7 ± 6.3^b^^∗∗^
Group 20	190.4 ± 5.5^b^^∗∗^

Each value is Mean ± S.E.M (*n* = 3). (^*∗*^) designates statistically significant difference from the respective group using Dunnett's multiple comparison test (^∗∗^*p* < 0.01). (*η*) designates statistically no significant difference from the respective group using Dunnett's multiple comparison test (*p* > 0.05). Group 11 = normal control, Group 12 = CCl_4_+ phenobarbitone control, Group 13 = CCl_4_+ phenobarbitone + 2.6 ml/kg/day Holyliv, Group 14 = CCl_4_+ phenobarbitone +5.2 ml/kg/day Holyliv, Group 15 = CCl_4_+ phenobarbitone + 2.6 ml/kg/day Heptaliv, Group 16 = CCl_4_+ phenobarbitone + 5.2 ml/kg/day Heptaliv, Group 17 = CCl_4_+ phenobarbitone + 2.6 ml/kg/day Icturn, Group 18 = CCl_4_ + phenobarbitone +5.2 ml/kg/day Icturn, Group 19 = CCl_4_ + phenobarbitone +2.6 ml/kg/day J-Deenar, Group 20 = CCl_4_+ phenobarbitone + 5.2 ml/kg/day J-Deenar.

## Data Availability

The data are all included within the paper.

## Abbreviations

CCL_4_: Carbon tetrachloride
ROS: Reactive oxygen species
PLFs: Polyherbal formulations
AST: Aspartate transaminase
ALT: Alanine transaminase
ALP: Alkaline phosphatase.
